# Preferential Occupancy of R2 Retroelements on the B Chromosomes of the Grasshopper *Eyprepocnemis plorans*

**DOI:** 10.1371/journal.pone.0091820

**Published:** 2014-03-14

**Authors:** Eugenia E. Montiel, Josefa Cabrero, Mercedes Ruiz-Estévez, William D. Burke, Thomas H. Eickbush, Juan Pedro M. Camacho, María Dolores López-León

**Affiliations:** 1 Departamento de Genética, Facultad de Ciencias, Universidad de Granada, Granada, Spain; 2 Department of Biology, University of Rochester, Rochester, New York, United States of America; Louisiana State University, United States of America

## Abstract

R2 non-LTR retrotransposons exclusively insert into the 28S rRNA genes of their host, and are expressed by co-transcription with the rDNA unit. The grasshopper *Eyprepocnemis plorans* contains transcribed rDNA clusters on most of its A chromosomes, as well as non-transcribed rDNA clusters on the parasitic B chromosomes found in many populations. Here the structure of the *E. plorans* R2 element, its abundance relative to the number of rDNA units and its retrotransposition activity were determined. Animals screened from five populations contained on average over 12,000 rDNA units on their A chromosomes, but surprisingly only about 100 R2 elements. Monitoring the patterns of R2 insertions in individuals from these populations revealed only low levels of retrotransposition. The low rates of R2 insertion observed in *E. plorans* differ from the high levels of R2 insertion previously observed in insect species that have many fewer rDNA units. It is proposed that high levels of R2 are strongly selected against in *E. plorans*, because the rDNA transcription machinery in this species is unable to differentiate between R2-inserted and uninserted units. The B chromosomes of *E. plorans* contain an additional 7,000 to 15,000 rDNA units, but in contrast to the A chromosomes, from 150 to over 1,500 R2 elements. The higher concentration of R2 in the inactive B chromosomes rDNA clusters suggests these chromosomes can act as a sink for R2 insertions thus further reducing the level of insertions on the A chromosomes. These studies suggest an interesting evolutionary relationship between the parasitic B chromosomes and R2 elements.

## Introduction

Transposable elements (TEs) appeared to be an oddity when they were first discovered by B. McClinctock in the 1950s, but now they are known in all sequenced genomes except *Plasmodium falciparum* [1]. TEs constitute a major percentage of all large eukaryotic genomes. Due to the huge variety of mobile elements – 22586 entries in RepBase Update [2] – it has been necessary to classify them on the basis of transposition mechanism and molecular structure (see RepBase Update). Within the retrotransposons (Class I), the R superfamily of non-LTR elements includes several members (e.g. R2 elements) which specifically insert into a highly conserved region of the 28S rRNA genes. R2 insertions were first observed in the fly *Drosophila melanogaster* [3] but are now known to be present in many arthropod species, such as *Bombyx mori*, *Apis melifera*, *Rhyncosciara americana* and *Triops cancriformis* [4]–[9] as well as in other animal phyla such as Chordata, Echinodermata and Platyhelmintes [10], [11].

R2 retrotransposable elements encode a protein with three domains: a reverse transcriptase (RT) occupying a central position, an endonuclease (EN) at the C terminus, and a DNA binding domain at the N terminus [12]. The protein encoding domains are flanked by two untranslated sequences (5′UTR and 3′UTR). Unlike most other retroelements, the 5′ UTR region of R2 does not contain promoter sequences, but instead encodes a self-cleaving ribozyme which allows R2 elements to be co-transcribed with the rDNA unit [13]. The 3′ UTR region of many R2 elements have poly(A) tails similar to other non-LTR elements [14].

The R2 insertion mechanism has been extensively studied in *B. mori* [15]–[18]. R2 inserts into rDNA units through cleavage of the 28S target site by an R2-encoded endonuclease, use of this cleaved end for target primed reverse transcription (TPRT) by its encoded reverse transcriptase, followed by second strand DNA cleavage and synthesis of the second DNA strand, and finally DNA repair. In many insects this mechanism produces variable length deletions at the 5′end of the R2 element [19]. These 5′-truncated copies serve as footprints of new insertion events [20], [21].

In all organisms, the many rDNA units maintain high sequence homogeneity (concerted evolution) by the recombinational process of unequal crossovers and possibly gene conversion (reviewed in [22]). The ability of R2 elements to escape this homogenization is explained in part by their high insertion rate [23]. While low levels of R2 are not strongly selected against because more rDNA units are encoded in the host genome than are needed for organism survival, high levels would be detrimental to the host [21]. A recent model proposed for the regulation of R2 elements suggests that organisms attempt to limit transcription to regions of the rDNA locus free of R2 insertions [24]–[26]. In most individuals in a population, large blocks of R2-free rDNA units are present, and thus R2 elements are excluded from transcription. However, the continuous reconfiguration of the rDNA locus by crossovers varies the number of R2 copies and their distribution leading eventually to R2 activity. In many insects 10–30% of the rDNA units are inserted by R2 elements, however, in other species the insertion level is lower [8], [26], [27]. Transcription of R2 elements has been found in many adult tissues of *D. simulans* [22] but most retrotransposition activity may take place during oogenesis and early embryo development [28].

Supernumerary B chromosomes were discovered at the dawn of cytogenetics [29], [30] and have been reported in about 15% of eukaryotic organisms. In contrast to standard (A) chromosomes, B chromosomes are dispensable, show a variable number among individuals, and do not always occur in pairs, thus do not follow Mendelian inheritance. In addition, they are frequently heterochromatic, do not recombine with A chromosomes, and may be deleterious for the organism carrying them. B chromosomes are most likely derived from A chromosomes, either intra- or interspecifically, but follow their own evolutionary pathway (for a recent review, see [31]). The evolutionary dynamics of B chromosomes usually follow a parasitic pathway, as was first suggested by Östergren [32] and later recognized by others [33]–[36]. Alternatively, Darlington [37] and White [38] suggested a heterotic nature for B chromosomes arguing that they could be beneficial at low numbers. However, such a beneficial effect of B chromosomes has only been shown in a few cases (e.g. [39], [40]).

In the grasshopper *Eyprepocnemis plorans*, B chromosomes begin as parasitic elements in newly invaded populations and rapidly increase in frequency through meiotic drive in females [41]–[43]. B meiotic drive is rapidly neutralized through the action of drive suppressor genes [41], [44], [45] and, during their subsequent near-neutral period, new parasitic B variants (recovering drive) may invade the population replacing the original B [42]. B chromosomes in *E. plorans* show a high mutation rate and thus over 50 B variants have been found [46], [47]. These variants mainly differ in the amount and distribution of their two major repeated DNA sequence constituents: a 180 bp tandem repeat (satDNA) and rDNA [48]. Recently, transcriptional activity of the rDNA in the B_24_ chromosome from the Torrox population has been inferred from the presence of nucleoli attached to the distal region of B chromosomes during the first meiotic prophase [49], [50] and rRNA transcripts showing sequence specificity to the rDNA in the B chromosome [51]. In the A chromosomes, the principal rDNA clusters are located on the X chromosome and the M_9_, S_10_ and S_11_ autosomes, but smaller clusters are also present in most remaining autosomes [52].

B chromosomes have been considered to be havens for TEs because B dispensability would permit TEs to escape from the action of natural selection [53]. This may explain why TE accumulation has been reported in B chromosomes from fish [54] and maize [55]. However in *E. plorans*, *gypsy* and *RTE* retrotransposons as well as *Mariner* transposons are rarely found in heterochromatin, thus are scarce in heterochromatic B chromosomes [56]. The fact that B chromosomes in this species show large amounts of rDNA, made it feasible that the distribution will be different for an rDNA specific TE like R2. Here we characterize the complete DNA sequence of R2 in *E. plorans* and analyze R2 abundance in the genome of B-carrying (B+) and B-lacking (0B) individuals in order to increase our understanding of the relationship between mobile elements and the *E. plorans* genome, in general, and B chromosomes, in particular. We found that B chromosomes are enriched in R2 elements, that R2 elements are transcribed in ovary and eggs but not in male tissues or embryos, and that different R2 5′ truncations patterns are found in natural populations providing evidence of recent retrotransposition activity. Finally, we adapt current models of R2 activity to the unique rDNA environment in *E. plorans*.

## Results

### Molecular characterization of R2 in E.plorans

**Figure 1:**
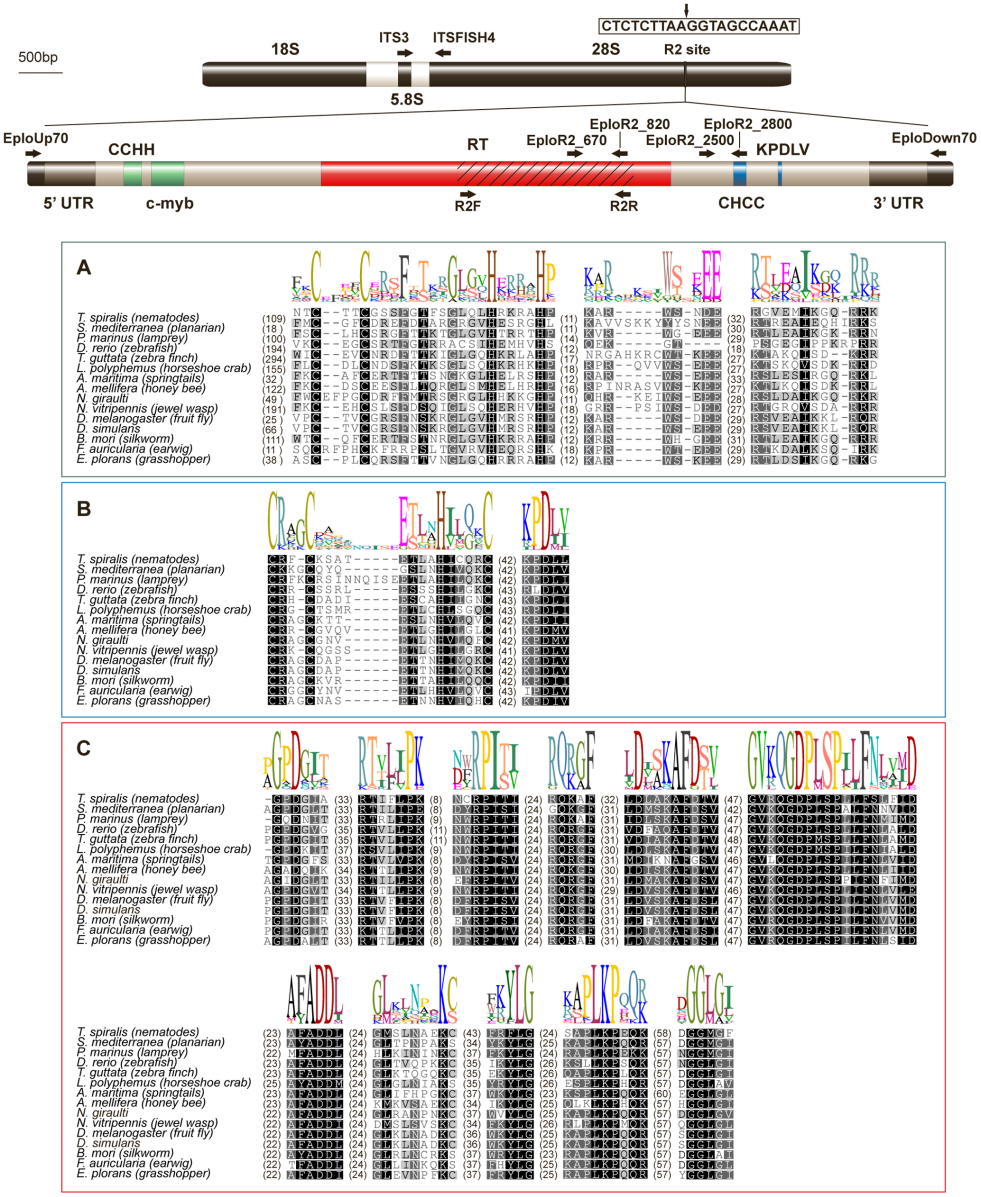
Diagram of the EploR2 insertion site, its molecular structure and alignment with other R2 elements. The rDNA transcription unit is represented above, with the R2 site indicated. The sequence of the insertion target (box) with a vertical arrow is indicating the exact site of insertion. In the diagram of the R2 element the striped area indicates EploR2_clon3, which was the first sequence obtained with the R2-F and R2-R degenerate primers. Arrows show position and orientation of primers used to clone the entire element and in the copy number estimation assay. The boxes show the alignment of the major domains of the R2 element with other R2 elements. Box A contains the CCHH and c-myb motifs of DNA-binding domain. Box B contains the CHCC motif and KPDLV of the endonuclease domain. Finally, box C shows the 9 motifs of the reverse transcriptase domain.

The general strategy to recover R2 elements using the degenerate PCR primers described in the literature [5] was not suitable for *E. plorans*. Therefore, we designed new degenerate primers, R2-F and R2-R, based on a more extensive set of R2 protein alignments from different organisms. R2-F and R2-R produced a pool of fragments ranging from 200 bp to 2 kb in length. Sequence analysis of 15 cloned PCR products from B+ males revealed five with an 859 bp insert showing sequence similarity with R2 elements in BLASTX and Repbase. To complete the sequencing of the R2 element in *E. plorans*, we employed EploUp70-F/EploR2_820-R and EploR2_670-F/EploDown70-R primer pairs (Fig. 1). From both PCR reactions we obtained fragments of the expected size (2 kb) that were subsequently cloned. Three of twelve clones analyzed from the 5′ end corresponded to the 5′ R2 region, and three of six clones from the 5′ end corresponded to the R2 element. Combining the sequences in these clones allowed us to elucidate the complete sequence of an R2 element from *E. plorans*.

As shown in Figure 1, the full-length R2 element in *E. plorans* (EploR2) is 3566 bp long (GenBank acc. no. JQ082370). As a non-LTR retrotransposon, EploR2 lacks long terminal repeats and contains a 206 bp 5′UTR region and a 234 bp 3′ UTR region. The 3′ UTR contains a canonical polyadenylation site (AATAAA) at nucleotide 3550, but it does not contain a terminal poly(A) tail as found in the R2 elements of some species. The sequence contains a single open reading frame encoding a polypeptide of 1041 amino acids.

To assess the similarity between EploR2 to R2 elements from other species, we aligned the EploR2 protein sequence with R2 proteins obtained from a wide range of animals (Fig. 1). The encoded protein contains all the domains found in the R2 proteins from other species: a DNA-binding domain in the N-terminal region, a reverse transcriptase domain in the middle, and an endonuclease domain in the C-terminal region. The amino-terminal region contained both the CCHH zinc finger and c-myb motifs involved in the recognition of the DNA target [17]. The carboxyl-terminal endonuclease domain contained both the Cys-His (CCHC) and KPDLV motifs [12].

### Copy number quantification

**Figure 2:**
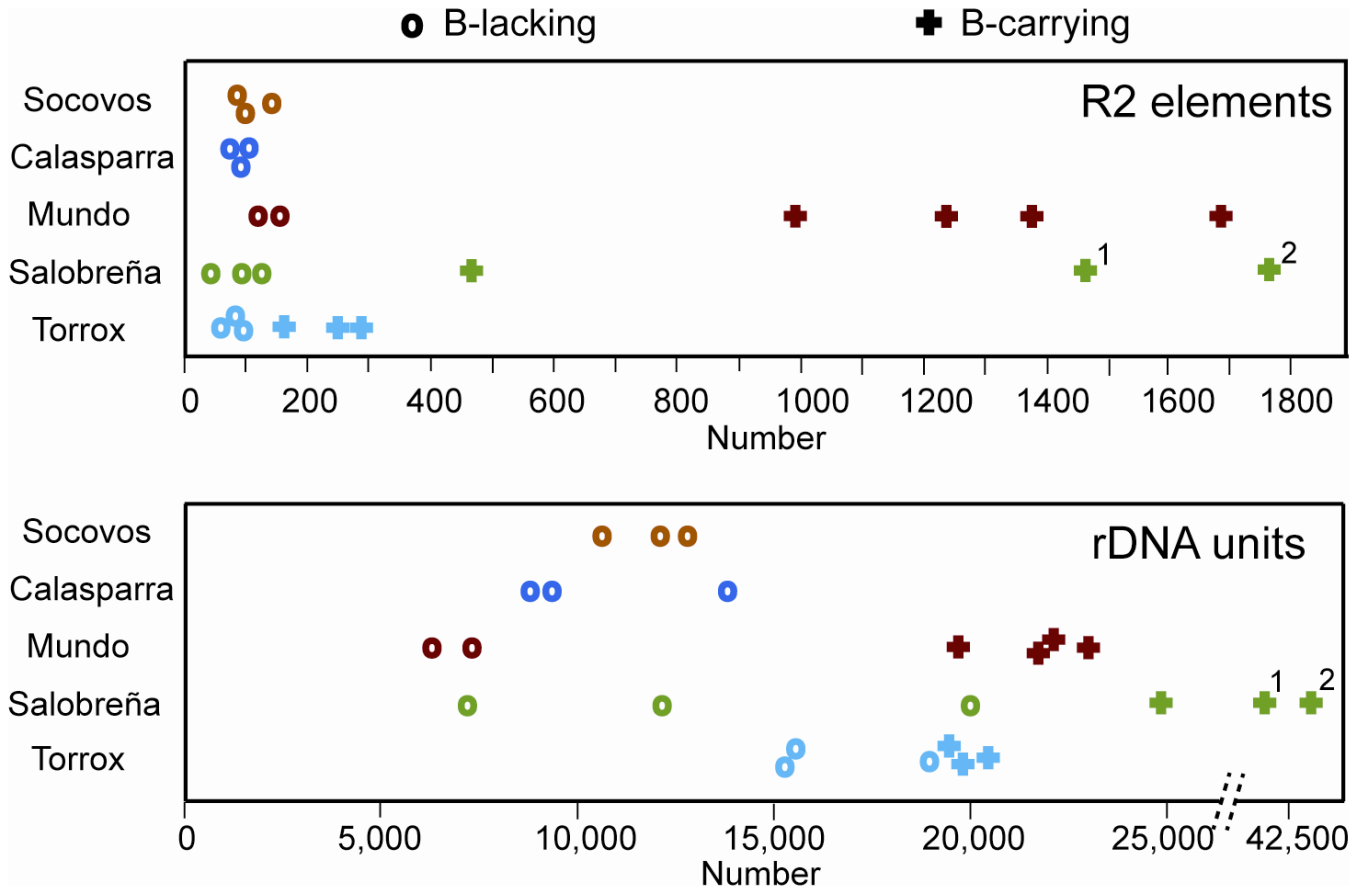
Total number of rDNA units and R2 elements determined for males carrying (+) or lacking (O) B chromosomes. Animals were obtained from five populations in Spain. Top panel, total number of R2 elements; Bottom panel, total number of rDNA units. ^1^, male with two B chromosomes; ^2^, male with a B chromosome that appears to have arisen from the fusion of two B chromosomes.

The presence of rDNA in the B chromosomes of *E. plorans* raised the possibility of differential R2 occupancy on the A and B chromosomes. Therefore, the total number of rDNA units and the total number of R2 elements were determined by qPCR in males from five populations differing in B chromosome frequency and type. These populations were: Socovos, no B chromosomes; Calasparra, low frequency of the B_1_ variant; and Mundo, Salobreña and Torrox, high frequency for the B_1_, B_2_ and B_24_ variants, respectively. As shown in Figure 2 (top panel), the 14 males without B chromosomes (0B males) from the different populations had from 66 to 146 copies of R2 with a mean value of 97 copies. This mean value is about twice that seen in populations of *D. melanogaster* and *D. simulans*, however, the 2.2 fold range is similar [26], [27]. The number of rDNA units in these same males varied from 6,400 units to 20,000 units with a mean of 12,200 (Fig. 2, lower graph). Significant differences in the mean number of rDNA units may exist between populations, because both males from the Mundo populations have about 7,000 units while all three males from the Torrox population averaged 17,000 units. The mean of 12,200 rDNA units across these 14 males in the five populations of *E. plorans* is approximately 50 times higher than the number of rDNA units seen in the two Drosophila species. Based on these determinations, less than 1% of the rDNA units on the A chromosomes of *E. plorans* are inserted with R2 elements compared with the mean insertion levels of 15% (*D. melanogaster*) and 20% (*D. simulans*) seen in the two Drosophila species.

Ten males with B chromosomes (B+ males) from three of the populations were also scored for the total number of R2 elements and rDNA units. Significantly greater numbers of R2 elements were detected in these B+ males. The range in R2 number in the B+ males also varied greatly between populations. B+ males from the Torrox population had from 160 to 290 copies of R2, while B+ males from the Mundo population had from 1000 to 1700 R2 copies. Averaged over the three populations, B+ males had a mean of 970 R2 elements or about 10 times the number found in 0B males. With respect to the number of rDNA units, B+ males were determined to have a mean of 25,600 (range 19,800 to 43,200), or twice the number of rDNA units seen in 0B males. These findings are consistent with the previous *in situ* demonstrations of large numbers of rDNA units on the B chromosomes [48], [52].

The 10-fold greater number of R2 elements but the only 2-fold higher number of rDNA units indicates that R2 elements are on average about 9-fold more abundant on the B chromosome rDNA units than on the A chromosome rDNA units. The abundance of R2 elements on the B chromosomes appeared to correlate with the estimated age of the B chromosome. The B variant in the Mundo population, B_1_, is considered the oldest variant in the Iberian Peninsula [57]. The mean number of rDNA units on this B variant can be estimated at 9,400 by subtracting the 12,200 unit average on the A chromosomes of 0B males from the mean 21,600 units observed in the animals carrying the B_1_ chromosome. However, the mean number of rDNA units on the B_1_ chromosome might be closer to 15,000 rDNA units, if the approximately 7,000 units on the two 0B males sampled from this population are representative of the entire population (Fig. 2). B_1_ chromosomes contain a mean of about 1,200 R2 insertions (mean 1,300 total R2s minus the 100 R2s on the A chromosomes). Therefore, B_1_ occupancy is about 8%.

The B chromosome variant in the Salobreña population has been defined as B_2_ [57]. Before estimating the number of rDNA units on this variant, it should be noted that two of the B+ males sampled from this population had two copies of the B_2_ chromosome (marked with superscripts in Fig. 2). The number of rDNA units on B_2_ chromosomes is estimated to be about 14,000 (i.e. the number of rDNA units in the B+ males minus 12,200 for the mean number of rDNA units on the A chromosome, and dividing the difference by two in the males with two B_2_ chromosomes). This estimate is similar to the estimate when the number of rDNA units on the A chromosomes is derived from the three 0B males tested from this population. The mean number of R2 elements on the B_2_ chromosomes is estimated at about 600, implying about 4.3% occupancy.

Finally, the B chromosome variant in the Torrox population, B_24_, has been described as arising recently [42], [57]. An estimated 7,000 rDNA units are on the B_24_ variants using the 12,200 units average on the A chromosomes of all populations, or a much smaller 3,000 rDNA units when using the large average number of rDNA units observed on the A chromosomes in the three 0B males sampled from this population. B_24_ chromosomes contain about 150 R2 insertions thus implying about 5% occupancy.

### Chromosome distribution of R2

**Figure 3:**
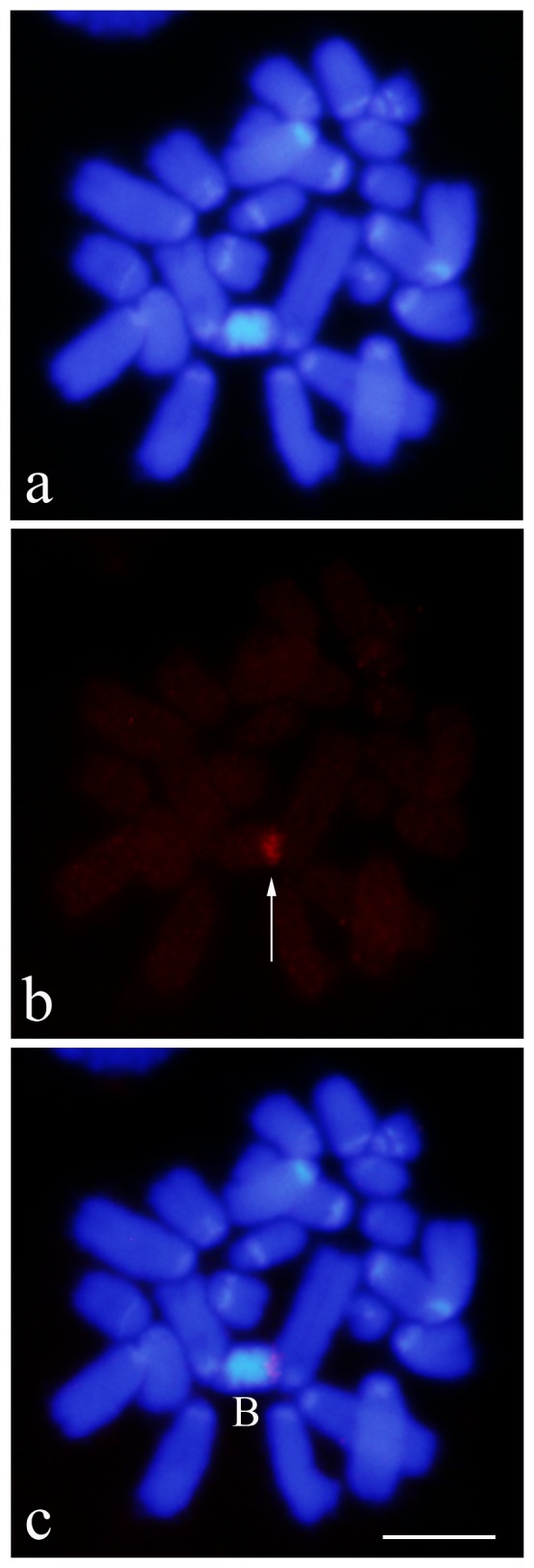
Mitotic metaphase belonging to an embryo from Torrox submitted to FISH with the EploR2 probe (red) and counterstained with DAPI. a) DAPI staining showing the presence of proximal and interstitial bright bands, corresponding to the location of the A+T rich 180 bp tandem repeat DNA specific of *E. plorans*. b) FISH with the EploR2 probe showing a single and large cluster (arrow). c) Merging of DAPI (a) and FISH (b) photographs showing the location of the R2 cluster (red) in the distal DAPI- region of the B_24_ chromosome (B), coinciding with rDNA location (Cabrero et al. 1999). Bar =  5 μm.

The greater concentration of R2 on the B chromosomes relative to the A chromosomes could be confirmed by direct physical mapping of R2 elements. 15 embryos from the Torrox population were subjected to fluorescence in situ hybridization (FISH) experiments using R2 sequences as probe. Hybridization was readily detected to the approximately 150 R2 elements on the distal DAPI^–^ region of the B_24_ chromosome, coinciding with location of the rDNA cluster in the B chromosome (Fig. 3). No signal was observed to the less than 100 R2 elements distributed over the A chromosomes. Potentially all A chromosomes in this population contain rDNA clusters with the largest clusters on chromosomes S_9_, S_10_, S_11_ and X [58].

### R2 Transcription and Retrotransposition Activity

**Figure 4:**
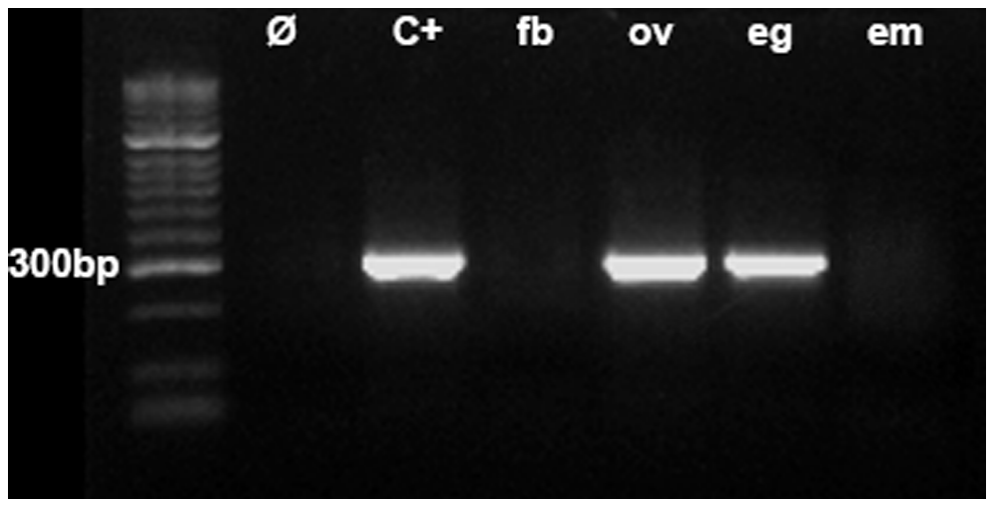
PCR analysis for the presence of R2 transcripts. R2 elements were observed in the cDNA obtained from ovaries (ov) and eggs (eg), but not in female somatic body (fb), embryos (em) and six male tissues (not shown). Ø =  Negative control (with no cDNA), C+ =  Positive control (genomic DNA).

In R2 active stock of *D. simulans*, R2 RNA transcripts are readily detected in embryos and many adult tissues of both males and females [24], however evidence has been obtained to suggest that R2 retrotransposition may only be occurring in ovaries and during the early development of embryos [28]. To determine if the R2 elements of *E. plorans* are also being transcribed, R2 transcripts levels were monitored in various individuals and tissues by PCR on cDNA obtained from total RNA. This was done in 12 B+ females (Torrox), nine B+ males (seven from Salobreña and two from Torrox), six different tissues from eight B+ males from Torrox (four with active B-nucleolar organizers (NOR) and four with inactive B-NOR), one gonad from a B+ female (Torrox), the eggs from an egg-pod (Torrox), and several embryos (Torrox). R2 element transcripts were not detected in males or from the dissected tissues of males (head, leg, wings muscle, testis, accessory gland, and gastric caeca) (data not shown). In the case of females, total bodies (lacking ovaries) and embryos also did not reveal transcripts, but R2 transcripts were detected in ovaries and eggs (Fig. 4). Thus unlike in Drosophila, R2 transcripts in *E. plorans* were not detected in most tissues of males and females, however, R2 transcription was detected in the tissue suggested to most likely support R2 activity (ovaries and eggs).

**Figure 5:**
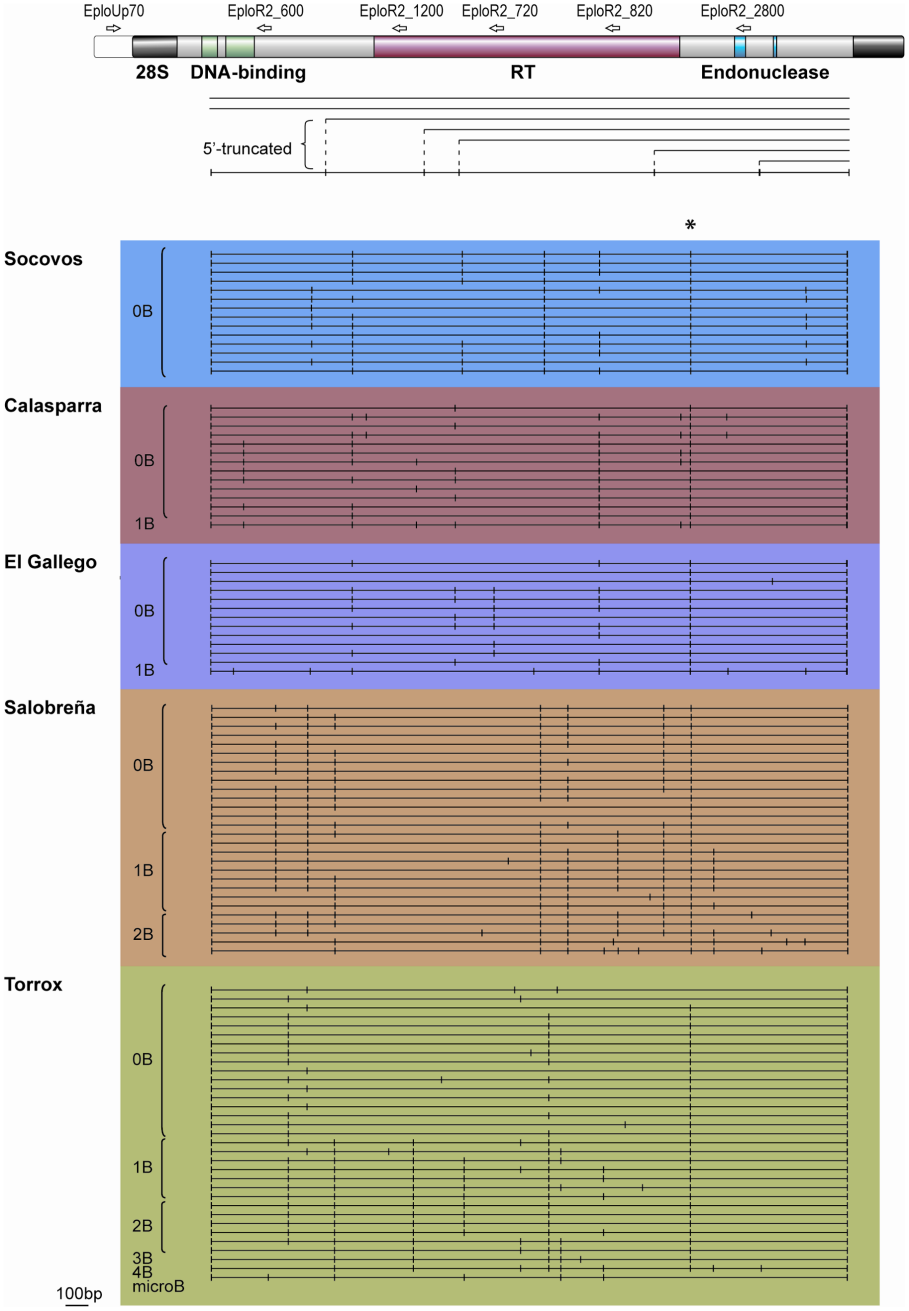
5′truncation profile of R2 elements in 0B and B+ (1–4) individuals from five populations of *E. plorans*. R2 element diagram shows the location of R2 PCR primers used to determine the length of 5'-truncated and full-length R2 elements. The collection of 5'-truncated and full-length R2 elements found in each individual has been represented in a summary diagram as in [59]. Each horizontal line represents the full-length R2 element in an individual, and the vertical lines indicate 5'-truncations. The asterisk indicates a 5′ truncation fixed or nearly fixed in all five populations. "microB" indicates 5' truncations observed in R2 copies contained in DNA microdissected from the B_24_ chromosome in Torrox.

To test for recent retrotransposition of R2 elements, we assayed for the presence of specific R2 copies in different individuals of various *E. plorans* populations. The approach used was developed in *D. simulans* [59] and is based on the finding that the R2 insertion mechanism frequently generates deletions at the 5′ end of the final integrated product. These 5′ truncations can be monitored by PCR amplification of genomic DNA using a forward primer that anneals to the 28S rRNA gene upstream of the R2 insertion site in combination with individual reverse primers that anneal to sequences spaced evenly throughout the R2 element (Fig. 5, top diagram). After PCR amplification of genomic DNA the products are separated by electrophoresis with each 5′ truncated R2 element giving rise to a unique length product. In *D. simulans*, different individuals from lines with active R2 retrotransposition display differences in their collection of 5′ truncated R2 elements (R2 5′ truncation profile) with some 5′ truncated copies unique to single individuals. Different individuals from a stock without R2 retrotransposition display identical 5′ truncation profiles indicating that recombinations within the rDNA loci do not generate new R2 truncations [59]. Thus the degree to which the pattern of 5′ truncated R2 copies differ among individuals within a population is an indication of the frequency of new R2 insertions in that population. After insertion, the length of time a 5′ truncated R2 copies survives in a population before being eliminated by recombination appears similar to that of full-length R2 elements [26].

The R2 5′ truncation profiles were determined for animals from five different *E. plorans* populations (Fig. 5). All individuals from these populations contained a limited number of truncations (range 2 to 9) in spite of the large number of R2 elements indicating that only a small fraction of the R2 retrotransposition events in *E. plorans* result in a 5′ truncation. Considering first the 13 to 14 animals tested from the three populations without frequent B chromosomes (Socovos, Calasparra and El Gallego), little evidence of recent R2 activity was obtained. In these populations most 5′ truncations were either present in all or a large fraction of the animals, suggesting the insertions had occurred long enough ago that they were now at high frequency in the populations. Some evidence for more recent R2 activity was found in the Calasparra and El Gallego populations where, in addition to 5′ truncation found at high frequency, 5′ truncations were present in only one or a few animals from each population.

In the two populations where B chromosomes were frequent (Salobreña and Torrox), the high frequency of 5′ truncations can be associated with either the A chromosomes or the B chromosomes. Evidence for recent R2 activity can readily be seen as truncations non-shared among animals. Direct evidence for the location of these 5′ truncations on the B chromosome in the Torrox population was obtained by conducting the PCR assays on microdissected B chromosomes obtained from one animal. As shown in the bottom line in Figure 5 isolated B chromosomes contained four high frequency truncations found only in B+ animals as well as one unique truncation. B-specific truncations can also be suggested for the B chromosomes in the El Gallego population, where five unique truncations are found in the one B+ animal tested.

Finally, R2 5′ truncations can also be used to monitor gene flow between populations. Only one 5′ truncation on the A chromosomes is old enough to be fixed or nearly fixed in all five populations (marked with an asterisk). Most high frequency 5′ truncations found in a population are unique to that population. However, Socovos, Calasparra and El Gallego populations share two 5′ truncations that are not found in the more southern Salobreña and Torrox populations.

## Discussion

The grasshopper *E. plorans* contains large numbers of rDNA units located on most of its A chromosomes, as well as high numbers of rDNA units on a parasitic B chromosome [58]. This contrasts with other insect species, such as Drosophila, where a smaller number of rDNA units are present at only a single locus. This difference in the number and distribution of rDNA units should have a profound effect on TEs, such as R2, which insert exclusively into the 28S rRNA genes of these rDNA units. Degenerate PCR primers were designed to highly conserved reverse transcriptase motifs of insect R2 elements to clone the R2 element of *E. plorans*. The *E. plorans* R2 element (EploR2) encodes all the domains typical of previously identified R2 elements. EploR2 encodes a single CCHH zinc-finger motif in its N-terminal domain, thus belongs to the R2-D clade, together with most other arthropod R2 elements [9], [60]. The EploR2 retrotransposition machinery appears more accurate than that of some other species as few 5′ truncated copies and few deletions of the upstream 28S rRNA sequences are generated during insertions. Such uniform insertions have been suggested to result from the self-cleaving ribozyme encoded at the 5′ end of the element cleaving the 28S/R2 co-transcript upstream of the 28S/R2 junction [61]. Consistent with this prediction, folding of the 5′ UTR sequence of EploR2 reveals a ribozyme that would cleave 13 nucleotides upstream of the junction (data not shown). A search for R2 transcription revealed R2 RNA in ovaries and eggs, suggesting R2 retrotransposition in females. This finding is similar to that in *D. simulans* where the highest levels of R2 retrotransposition activity has been shown to occur in the female germ line or during early embryo stages [28].

### An abundance of rDNA units but only low levels of R2 insertions

The mean number of rDNA units on the A chromosomes of *E. plorans* (i.e. in 0B individuals) was estimated to be 12,200. This value is about 50 times higher than the estimate of approximately 200 units in species of Drosophila [26], [27] and 4–12 times larger than estimates in other orthopterans. For example, Schäfer and Kunz [62] estimated 3,300 rDNA units in *L. migratoria* and Parkin and Butlin [63] estimated 1000 rDNA units in *C. parallelus*. One possible reason for the large number of rDNA units in *E. plorans* relative to other species is that *E. plorans* contains rDNA clusters on all A chromosomes, *C. parallelus* carries rDNA clusters on only two (or three) chromosome pairs, and *L. migratoria* on three pairs [64]. It will be possible to test this explanation, as populations of *E. plorans* from the far east, e.g. Dagestan, as well as different subspecies, such as *E. plorans meridionalis*, have rDNA clusters on only two A chromosomes [65]. Because *E. plorans* has also been estimated to have a larger genome than most insects, the fraction of the genome represented by rDNA (1.3%) is similar to that in *L. migratoria* (1%) and even *D. melanogaster* (0.9%) [62].

The mean number of R2 insertions on the A chromosomes of *E. plorans* is about 100. Thus the fraction of the rDNA units on the A chromosomes that are inserted with R2 elements is only 0.9% (range 0.4–2.0%). This range of insertion levels is similar to those found in a population study of the tadpole shrimp, *Triops cancriformis*, where R2 insertions were estimated at 0.5–5% of the rDNA units [9]. However, the level of R2 insertion in *E. plorans* and *T. cancriformis* is much lower than that in *D. melanogaster* and *D. simulans* where insertion levels typically range from 10 to 30% of the rDNA units [26], [27].

The total number of R2 insertions in a species is a balance between the rate of new retrotransposition events and the rate of net loss of elements by the recombination events (crossovers) that give rise to the concerted evolution of the rRNA genes [26]. Determination of these rates can only be obtained by detailed studies of element gain and loss over extended periods similar to those that have been conducted in Drosophila [20], [21], [59], [66]. However, a rough estimate of R2 turnover and thus R2 activity can be obtained by comparing the extent to which the pool of individual R2 elements differ between individuals in a population [59]. Comparison of the 5′ truncation profiles of individuals from five populations of *E. plorans* revealed a rate of R2 element turnover considerably below that found for populations of *D. simulans*. In *D. simulans* virtually all of the individual 5′ truncated copies of R2 are rare (i.e. unique to an individual or present in only a low percentage of the population) [25]. In *E. plorans* on the other hand (Fig. 5), most 5′ truncated R2 copies are broadly shared between individuals in a population with only a small fraction of rare copies. Thus the rate of R2 activity in *E. plorans* appears to be significantly lower than that of *D. simulans*.

Why would there be lower rates of R2 insertions in a species like *E. plorans* that have high numbers of rDNA units than in a species like *D. simulans* with much lower numbers of rDNA units? One formal possibility is that *E. plorans* needs its very large number of rDNA units to be transcribed, therefore, few can be spared for R2 insertion. The second possibility is that control over R2 activity is more effective in *E. plorans* than in *D. simulans*.

The first explanation appears unlikely. Indeed this model can be directly excluded since, as mentioned previously, *E. plorans plorans* from the east and *E. plorans meridionalis*, with rDNA clusters on only two chromosomes, are confirmed to have many fewer rDNA units. More importantly all eukaryotes have been found to contain many more rDNA units than are needed for transcription. This excess is derived from the crossovers that are responsible for concerted evolution of the rDNA units continually expanding and contracting the size of the rDNA clusters of individuals in a population. Because clusters with less than the minimal number of units needed for maximum fitness are eliminated from the population, the mean number of rDNA units for the population is much greater than the number needed for transcription [21]. The fraction of rDNA units being actively transcribed has been estimated at no more than 50% of the units in fast growing species with small genomes such as yeast [67] to as low as 15% of the total available rDNA units in Drosophila [68]. Thus, while *E. plorans* may need many more rDNA units than *D. simulans*, it is likely that *E. plorans* contains a large excess of rDNA units.

The second explanation for low levels of R2 elements in *E. plorans*, that this species is better able to control R2 retrotransposition events, appears more likely. This control could be direct, such as via small RNA pathways that prevent the production or promote the degradation of R2 RNA. However, because R2 RNA is co-transcribed with the rDNA unit, control over its activity may not be so direct. In an analysis of R2 activity in *D. simulans*, control over R2 transcription and retrotransposition activity mapped to the rDNA locus itself. However, R2 activity did not correlate with the size of the locus, the number of R2 insertions, or the fraction of inserted units in the locus [24], [25]. Instead R2 activity was correlated with the absence of a sizable region in the locus free of R2-inserted rDNA units. Based on these findings, we proposed a population genetic model for the frequency of R2 retrotranspositions in *D. simulans* that was able to explain the empirical data collected from the populations [26]. The model assumes that an estimated 40-unit region of the locus is selected for transcription at each generation. Second, that components of the rDNA transcriptional machinery can differentiate between R2-inserted and uninserted rDNA units,and that the 40-unit region of transcription is centered on the region with the lowest number of R2-inserted units. Selection in this model is thus not against chromosomes with the highest total number of R2 insertions, but rather against chromosomes in which R2 elements are located in the region of the locus that is transcribed. As a consequence high levels of R2 insertion can accumulate in the species through their insertion into regions that are not transcribed, typically the edges of the locus [26].

A significant, but simple, change to the transcription domain model is needed for it to explain the low fraction of R2 inserted units in *E. plorans*. If *E. plorans* transcriptional apparatus is less able to differentiate R2-inserted from uninserted rDNA units, then the selection of the transcription domain at each generation will be more random. As a result a large fraction of the units in the rDNA clusters will be transcribed at some point over multiple generations. Under this assumption the rate of R2 retrotransposition would directly correlate with the frequency of R2 insertions, and thus selection will most efficiently eliminate those chromosomes with the highest level of insertion. It should be noted that negative selection in this model is not a result of the individual not having sufficient numbers of uninserted rDNA units to be transcribed, but rather with the transcription of rRNA genes that are inserted and thus the production of defective rRNA. In other words, with no region within the rDNA loci where R2 elements can hide, much lower levels of R2 insertion would be expected for the species. This model can thus explain the apparent paradox of an organism with greater numbers of rDNA units and thus many more sites of R2 insertion, yet be selected to have lower numbers of inserted units than an organism with fewer rDNA units.

### The B chromosomes of E. plorans

The B chromosomes of *E. plorans* also contain high numbers of rDNA units. The three B variants assayed in this report encoded from 3,000 to 7,000 units in the case of B_24_ or 10,000 to 15,000 units in the cases of B_1_ and B_2_. These values are in general agreement with previous estimates using FISH [48]. The number of R2 elements on these B chromosomes varied widely: ∼150 on B_24_, ∼600 on B_2_, and ∼1,200 on B_1_. Thus the fraction of the B chromosome rDNA units occupied by R2 was from two to 10 times higher than the rDNA units on the A chromosomes.

The absence of functional genes and thus selection against insertions makes B chromosomes an excellent location for the accumulation of TEs. Indeed, several authors have shown an accumulation of TEs on B chromosomes [69], [70] or on sex chromosomes that have few essential genes, e.g. the Y chromosome of *D. miranda* [71]. Interestingly, this accumulation of TEs on the B chromosomes was not found for the *gypsy* and *RTE* retrotransposons or the *Mariner* transposons of *E. plorans* [56]. However, in these cases the cytogenetic and molecular data suggested these elements were most abundant in the euchromatic regions of the A chromosomes, and thus are less likely to insert in 180 bp satellite DNA and inactive rDNA that are the predominant components of the B chromosomes.

The accumulation of R2 on the B chromosomes because there is no selection against their insertion can also explain the distribution of R2 in *E. plorans*. Based on DNA methylation and histone hypoacetylation studies, the rDNA units on the B chromosomes of *E. plorans* are typically inactive. Thus the B chromosomes would act as sinks of no return for R2 element retrotransposition events originating from the A chromosomes. Consistent with this model the B_1_ variant, which is considered the oldest variant in the Iberian Peninsula [57], showed the highest proportion of rDNA clusters occupied by R2. Surprisingly, the B_24_ variant, which arose more recently in the Torrox population [42], [57], showed higher R2 occupancy than its ancestor variant (B_2_). A possible reason for this faster accumulation of R2 in the B_24_ chromosome is that it expresses its rDNA units in about half of the males from the Torrox population [49]–[51], whereas expression of the rDNA units on the B_1_ and B_2_ variants is seldom observed [72], [73]. It is thus conceivable that this increase in the expression of the B_24_-rDNA has increased the activity of R2 in this population and, since the B carries the largest rDNA cluster in the genome, it actually represents the most likely target for R2 reinsertion. Consistent with this model the Torrox population has the greatest number of R2 5′ truncations that arose recently (i.e. unique to one individual) (Fig. 5).

There are over 50 different B chromosome variants reported in Spanish populations of *E. plorans* that appear to have arisen through chromosome rearrangements (e.g. inversions, deletions and translocations) involving breakpoints within the lightly C-banded distal region of the ancestral B chromosomes [46]. This distal region harbors the rDNA units [48], [58]. However, in only one variant, a Moroccan population, does it appear that the rDNA units have been deleted [47]. Why are these rDNA units being maintained, if these units are not contributing to the production of rRNA?

One likely explanation is that the rDNA units either promote the initial meiotic drive or the long-term propagation of the B chromosomes in a population. Because in the Spanish populations of *E. plorans* studied here all A chromosomes contain rDNA units, these units may be involved in chromosome pairing, alignment on the metaphase plate, or movement to either daughter cell during mitosis or meiosis. Such a model would also explain why this species has such an extraordinary number of rDNA units. A second more speculative model for the persistence of rDNA units on the B chromosomes of *E. plorans* is that by serving as a sink for R2 insertions the B chromosomes confer a advantage to the host. For example, if the rDNA units are equally distributed between the A and B chromosomes, then only half of new R2 retrotransposition events will occur in a potentially functional gene. Whatever the reason for the large number of rDNA units on the A and B chromosomes of *E. plorans*, this species serves as a fascinating model system to study the propagation of parasitic chromosomes and transposable elements.

## Materials and Methods

### Animals, cytological characterization of B chromosome variants and B-NOR activity

Adult specimens of *Eyprepocnemis plorans* were collected in Spanish populations at Torrox (Malaga), Salobreña (Granada), Calasparra (Murcia), El Gallego (Albacete), Mundo (Albacete) and Socovos (Albacete). No specific permits were required for this field study. The locations sampled were not privately owned or protected in any way, and this field study did not involve endangered or protected species. Adults were bred in the laboratory in order to obtain embryos. Egg pods were incubated at 28°C for twelve days, after which embryos were fixed in 3∶1 ethanol-acetic acid for cytogenetic studies as described in [74]. Males were anaesthetized in ethyl acetate vapours and testes were dissected out and fixed in freshly prepared 3∶1 ethanol-acetic acid. Males from Torrox were also dissected to obtain six different body tissues (head, leg, wing muscle, testis, accessory gland, and gastric caeca), which were frozen in liquid nitrogen and stored at -80°C until use. Females were injected with 0.1 ml of 0.05% colchicine in insect saline solution 6 h prior to anesthesia, dissection, and fixation of ovarioles in 1∶3 acetic acid-ethanol for cytogenetic purposes. Adult bodies, embryos, or dissected tissue were frozen in liquid nitrogen and stored at −80°C for DNA and RNA isolation. The number of B chromosomes in each individual was determined in 2% lactopropionic orcein squash preparations of two testis tubules or one ovariole. To visualize the nucleoli attached to B chromosomes in diplotene cells, testis follicle preparations were analysed by silver staining as indicated in [75]. Preparations were also stained with 1% Giemsa to differentiate chromatin (blue-green) from nucleoli (brown). For each test at least 20 diplotene cells per male were photographed with an Olympus digital camera (DP70).

### DNA and RNA isolation

Genomic DNA from adult individuals was extracted using the Gen Elute Mammalian Genomic DNA Miniprep Kit (Sigma-Aldrich) following the manufacturer's recommendations. Total RNA was isolated from 30 mg of individual frozen bodies using the Real total RNA spin plus kit (Durviz) following the manufacturer's protocol including DNase I treatment. RNA extractions of six different tissues from males, and from female carcasses lacking gonads, ovaries, eggs, and embryos were performed using RNeasy Lipid Tissue Mini Kit (Qiagen), following the manufacturer's recommendations. RNA was later submitted to a second treatment with 20 units of DNase I to ensure complete removal of contaminating genomic DNA. Quantity and quality of gDNA and RNA were measured with Tecan's Infinite 200 NanoQuant and in a denaturing agarose gel to ensure the absence of RNA degradation.

### B chromosome microdissection

Fifteen B chromosomes were microdissected from spermatocytes in one B24-carrying individual from the Torrox population. Procedures for chromosome preparation and microdissection assay were described in [76]. DNA of microdissected B chromosomes was amplified with the GenomePlex Single Cell Whole Genome Amplification Kit (WGA4-Sigma) following the manufacturer's recommendations.

### PCR amplification, DNA cloning and sequence analyses

To amplify R2 in *E. plorans* we designed degenerate primers, R2-F and R2-R, anchored on conserved segments of the reverse transcriptase domain, using the CODEHOP software [77]. Information about all primers is shown in Table 1. PCR experiments were performed in a 50 μl reaction mixture containing 50 ng of genomic DNA, 0.2 μM of each primer, 0.2 mM dNTPs, 1.25 mM MgCl_2_, and 1 unit of Taq polymerase (MBL). PCR conditions were as follows: initial denaturation 5 min at 94°C, 30 cycles of 1 min at 94°C, 1 min at 50°C, and 2 min at 72°C, and a final elongation for 10 min at 72°C. PCR products were visualized in a 1% agarose gel and were cleaned with the GenElute PCR Clean-up kit (Sigma-Aldrich). All amplified fragments were cloned into *pCR2.1-TOPO vector* (Invitrogen). Plasmid DNA was obtained with the Gen Elute Mammalian Genomic DNA Miniprep kit (Sigma), and clones were sequenced at Macrogen Inc. (Seoul, Korea). DNA sequences were analyzed with BioEdit [78] and Geneious Pro 4.8.5 (Biomatters Ltd.).

**Table 1:** List of primer with their sequence and the method where they have been used.

Primer name	Sequence (5′ -> 3′)	Method
R2-F	AGCGCCCACAGGATTTYCGNCCNAT	*C*
R2-R	AGGGCGAACATCCGCTGYTGBGGT	*C*
EploUp70-F	TGCCCAAGTGCTCTGAATTGTC	*C*, *TP*
EploDown70-R	AGATAGGGACAGTGGGAATC	*C*
EploR2_2800-R	CGAGATGGTAGAGCACTAATC	*TP*, *CN*, *E*
EploR2_2500-F	CAAGTCCCTCCATGCTCGCCA	*TP*, *CN*, *E*
Eplo R2_820-R	GGTGCCCGCTTAATGATGTCC	*C*, *TP*
Eplo R2_720-R	GGAGTGGGCCATCGCCAGATC	*TP*
Eplo R2_1200-R	GTAATCGGTGCCCACAGATC	*TP*
EploR2_670-F	AAGGTCGACAACAGTGTCATC	*C*
Eplo R2_600-R	AATAGAGTCCAACGTCCGGTC	*TP*
ITSFISH4	ATATGCTTAAATTCAGCGGG	*CN*
ITS3	GCATCGATGAAGAACGCAGC	*CN*

*C*: Initial cloning of element; *CN*: Copy number estimation; *E*: Expression analysis; *TP*: 5′ Truncation profile.

In order to characterize the complete sequence of R2 in *E. plorans*, we designed divergent primers, two of which were anchored in the *E. plorans* reverse transcriptase partial DNA sequence obtained, the EploR2_670-F and EploR2_820-R primers, and the other anchored in the 28S rRNA gene described in Gomphocerine grasshoppers (EploUp70-F and EploDown70-R) (see positions in Fig. 1). Thereby, we expected to amplify the 5′ region with the EploUp70-F/EploR2_820-R primer pair and the 3′ region with EploR2_670-F/EploDown70-R primer pair. PCR reactions were prepared as described above and were performed as follows: 2 cycles of 1 min at 97°C, 2 min at 55°C, and 3 min at 72°C, following for 28 cycles of 1 min at 94°C, 1 min at 60°C, and 3 min at 72°C, and final cycle of 1 min at 94°C, 1 min at 60°C and 10 min at 72°C. Amplifications using the EploUp70-F/EploR2_820-R and EploR2_670-F/EploDown70-R primer pairs yielded a pool of different length fragments. In both cases, the largest amplicons observed by electrophoresis (about 2 kb length) were purified with the GenElute Gel Extraction Kit (Sigma-Aldrich), cloned, and sequenced. Clone similarity to TEs was tested using BLAST and BLASTX tools [79] at the NCBI site. To confirm and refine the identity of the elements found within the vast families of non-LTR retrotranspons, we tested cloned DNA sequences with the CENSOR online software [2].

The molecular structure of the *E. plorans* R2 was determined by alignment with R2 elements from other species. From the Repbase Update database (http://www.girinst.org/repbase/update/index.html), we downloaded the aminoacid sequence of R2 elements from *Trichinella spiralis* (R2-1_TSP; nematodes), *Schmidtea mediterranea* (R2-1_SM; freshwater planarian), *Petromyzon marinus* (R2-1_PM; lamprey), *Danio rerio* (R2Dr; zebrafish), *Taeniopygia guttata* (R2-1_TG; zebra finch), *Limulus polyphemus* (R2_LP; horseshoe crab), *Anurida maritima* (R2_AM; springtails), *Apis mellifera* (R2Amel; honey bee), *Nasonia giraulti* (R2C_NGi), *Nasonia vitripennis* (R2B_NVi; jewel wasp), *Drosophila melanogaster* (R2_DM; fruit fly), *Drosophila simulans* (R2_DSi), *Bombyx mori* (R2_BM; silkworm) and *Forficula auricularia* (R2_FA; earwig). The alignment was done with the alignment tool implemented in Geneious Pro 4.8.5 (Biomatters Ltd.).

### Fluorescent in situ hybridization (FISH)

Chromosome preparations from 15 embryos from the Torrox population, thus carrying the B_24_ chromosome, were performed according to [74]. Slides were dehydrated in a series of 70%, 90%, and absolute ethanol and then incubated in an oven at 60°C overnight. Insert from EploR2_clon3 labeled by nick translation with tetramethylrhodamine-11-dUTP (Roche) was used as probe. Fluorescent hybridization signals were directly detected. About 250 ng of DNA probe was used in each FISH experiment following the technique described in [52]. Chromosomes were counterstained with DAPI (4′, 6 diamidino-2-phenylindole) and the analysis of chromosome preparations was made using an Olympus BX41 microscope for fluorescence, and photographs were taken with an associated DP70 cooled camera. Images were optimized for bright and contrast with THE GIMP freeware.

### Copy number estimations

The analysis of copy number for the R2 element and the total number of rDNA units in the *E. plorans* genome was performed by real-time quantitative PCR (qPCR). DNA was quantified using PicoGreen dsDNA Quantitation Reagent (Molecular Probes) in a fluorometer TBS-380, according to the manufacturer's recommendations. qPCR assays contained 10 μl of 2× SensiMixPlus SYBR (Quantace), 0.25 μM of each primer, and 10 ng of genomic DNA in a 20 μl final volume. The quantification of R2 copy number was done with the EploR2_2500F and EploR2_2800R primers which yield a 295 bp amplicon, and rDNA copy number was analysed with the ITS3/ITSFISH4 primers which amplified a 376 bp fragment.

R2 element and rDNA unit sequences were amplified with the same PCR programs, differing only in annealing temperature. The PCR program consisted in a denaturation step at 95°C for 10 min, and 40 cycles of 15 s at 95°C, 15 s at 58.2°C (R2), 61°C (rDNA), 72°C for 30 s, and a final step at 72°C for 5 min. Real-time qPCR assays were run in a Chromo4 (Biorad) thermocycler using Opticon Monitor v3.1 software to export raw data and LingRegPCR 11.0 programme to analyse them. Each sample was amplified in triplicate, and product specificity was verified through generation of a melting curve following Real-time PCR assay. The calibration factor was obtained with a serial dilution of two clones containing the region to be amplified, EploR2_2000.1 and EploITS_1.6.

Copy number estimations were performed through analysis with LingRegPCR software, following the method described by [80]. We estimated copy number of R2 and rDNA sequences on the basis of C values in *E. plorans* (10160 Mb) and recent estimates of the size of X and B chromosomes [81]. Therefore, 2C value in 0B males was calculated as twice the C value reported in [81] minus the DNA content of an X chromosome, since sex chromosome determination is X0 in *E. plorans* males. In B+ males, we estimated 2C value by also adding the DNA content of each B chromosome variant reported in [81].

### R2 expression and 5′ truncation profile

cDNA was obtained with SuperScript III First-Strand Synthesis SuperMix kit (Invitrogen), using random hexamer primers according to the manufacturer's recommendations. R2 retrotransposon expression was investigated by PCR amplification of the cDNA. The reaction mixtures contained 5 μl 2× SensiMix SYBR Mastermix (SensiMix SYBR Kit, Bioline), 0,7 μM each forward (EploR2_2500-F) and reverse (EploR2_2800-R) primer and 10 ng cDNA, in a final volume of 15 μl. PCR assays were run in the Chromo4 Real Time PCR thermocycler (BioRad) with the following conditions: an initial denaturation at 95°C for 10 min, 30 cycles of 94°C for 30 s, 55°C for 30 s, 72°C for 30 s, and a final step at 72°C for 8′. After the runs, a dissociation step was performed to check the specificity of the reaction. Each run included a negative control, lacking cDNA, to dismiss contamination of reagents and a positive control with genomic DNA to test the suitability of the primers. Each sample was amplified in triplicate. To confirm that the PCR amplification products corresponded to R2, the products were visualized in 1.5% agarose gel electrophoresis, cleaned with Gen Elute PCR Clean-Up Kit (Sigma), cloned into the TOPO TA vector (Invitrogen), and sequenced (Macrogen).

The analysis of R2 retrotransposition activity based on 5′truncation patterns was performed separately on individuals from five populations: three populations from the Southeast of the Iberian Peninsula (Calasparra, El Gallego and Socovos) where B chromosomes are absent or show very low frequency, and two populations from the South (Salobreña and Torrox) where B chromosomes are frequent. The 5′ truncation patterns were obtained by PCR amplification using the EploUp70-F primer, annealing upstream of the R2 element insertion site, coupled with various R2-specific primers: EploR2_600-R, EploR2_1200-R, EploR2_720-R, EploR2_820-R, EploR2_2800-R. These primers anneal at 561, 1356, 1551, 2200 and 2747 positions downstream of the insertion site, respectively. Amplifications were performed with 50 ng of genomic DNA and PCR conditions were: 2 cycles of 1 min at 97°C, 2 min at 55°C, and 3 min at 72°C, following for 28 cycles of 1 min at 94°C, 1 min at 60°C, and 3 min at 72°C, and final cycle of 1 min at 94°C, 1 min at 60°C, and 10 min at 72°C. PCR products were visualized in a 2% agarose gel, and the photographs were taken with a Gel Doc XR System (BioRad Laboratories, Inc). Photographs were analyzed with Quantity One v4.6.3 (BioRad Laboratories, Inc).
